# A proposal to account for the stimulus, the mechanism, and the mediators released in exercise-induced bronchoconstriction

**DOI:** 10.3389/falgy.2023.1004170

**Published:** 2023-11-06

**Authors:** Sandra D. Anderson, Pascale Kippelen

**Affiliations:** ^1^ Faculty of Medicine and Health, University of Sydney, Sydney, NSW, Australia; ^2^ Division of Sport, Health and Exercise Sciences, College of Health, Medicine and Life Sciences, Brunel University London, Uxbridge, United Kingdom

**Keywords:** exercise, asthma, bronchoconstriction, mast cells, inflammatory mediators

## Abstract

Exercise induced bronchoconstriction (EIB) describes the transient narrowing of the airways that follows vigorous exercise. It commonly occurs in children and adults who have asthma and in elite athletes. The primary stimulus is proposed to be loss of water, by evaporation, from the airway surface due to conditioning inspired air. The mechanism, whereby this evaporative loss of water provokes contraction of the bronchial smooth muscle, is thought to be an increase in osmolarity of the airway surface liquid. The increase in osmolarity causes mast cells to release histamines, prostaglandins, and leukotrienes. It is these mediators that contract smooth muscle causing the airways to narrow.

Exercise-induced bronchoconstriction (EIB) describes a transient narrowing of the airways that follows vigorous exercise. It most commonly occurs in children and adults with asthma and in elite athletes (1, 2). From early controversies about the primary stimulus of EIB (i.e., loss of water vs. loss of heat from the airways as a result of conditioning of large volumes of inhaled air during exercise-hyperpnea) (3) to the establishment of the mechanism of EIB (i.e., bronchial smooth muscle contraction in response to an osmotic-driven inflammation in the lower airways) (4) and subsequent identification of specific mediators involved (i.e., histamines, prostaglandins, and leukotrienes) (5–10), significant progress has been made in the field over the last 50 years. Herein, we summarize (using select illustrations) our current understanding of the stimuli, mechanisms, and mediators released in EIB.

Exercise-induced bronchoconstriction (EIB) is usually identified by measuring peak expiratory flow (PEF) or preferably forced expiratory volume in 1 s (FEV_1_). These lung function measurements are made immediately before exercise and for at least 10–20 min after exercise. Surrogates for exercise, such as dry air hyperpnea and osmotic agents (e.g., hypertonic saline or mannitol), may be used to identify the potential for EIB (11). When using exercise to identify EIB, it is important to consider exercise modality, as exercise by running is more potent than exercise by walking, swimming, or cycling (12). The intensity and duration of the exercise are also important (Figures 1A,B) (13). The exercise intensity needs to be sufficient to increase the participant's heart rate within a couple of minutes to 85% or more of their predicted maximum heart rate for exercise (14). This intensity should be sustained for at least 6 min in adults and 4–5 min in children. A nose clip is used to ensure that, during exercise, the subject is breathing by mouth. To reduce the risk of a false negative test, the inspired air needs to be “dry” with a water content of less than 10 mg H_2_O per liter. This value is achieved when the inspired air temperature is 23°C or less and its relative humidity is 50% or less (15). Medical air inhaled from cylinders usually meets these conditions. EIB is identified by documenting a reduction from the pre-exercise PEF or FEV_1_ of 13% in children and 10%–15% in adults (16, 17). These values are based on observations made in healthy non-asthmatics performing exercise under the same conditions. For those currently receiving treatment for asthma, appropriate times since they last took a medication must be documented, together with details of dose and duration of treatment (18).

**Figure 1:**
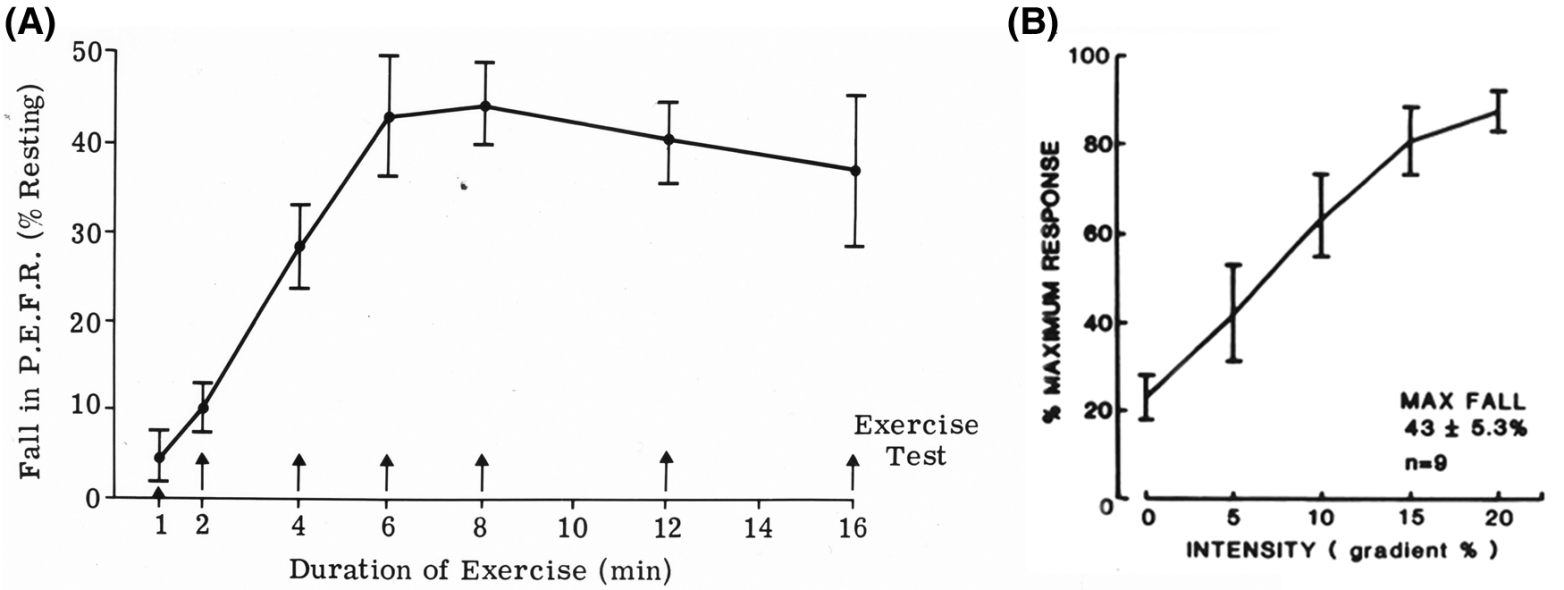
(**A**) Effect of the duration of exercise on the reduction in peak expiratory flow rate induced by treadmill running at constant speed and slope. Each point represents the mean of tests in 10 participants with asthma who performed each duration on a separate occasion. Modified from Silverman and Anderson (13). (**B**) Effect of gradient (workload) on the reduction in peak expiratory flow rate induced by treadmill running at constant speed for 6 min. Each point represents the mean of tests in nine participants who performed each gradient on a separate occasion. Modified from Silverman and Anderson (13).

Under most climatic conditions, when air is inspired, it needs to be heated to body temperature and fully humidified before entering the lower airways. At resting levels of ventilation, the conditioning of the inspired air to 37°C and full saturation of 44 mg H_2_O per liter can be achieved with nasal breathing. As ventilation increases during exercise, there is a need to reduce airway resistance, which is achieved by switching from nose breathing to mouth breathing. As a result of this switch, and due to the larger volume of air entering the respiratory tract, the lower airways are needed to fully condition the inspired air (19).

Mathematical models of the human airways suggest that the cumulative volume of airway surface liquid in the first 6–10 generations of lower airways is likely less than 1 ml. This value is based on the surface area of these generations and the depth of the airway surface liquid or periciliary fluid (19, 20). This fluid contains sodium and chloride ions and has an osmolarity of about 300 milliosmoles (similar to blood osmolarity). It has been proposed that, during exercise, the rate of water lost by evaporation from these generations due to conditioning the inspired air exceeds the rate of water return to the airway surface (Figure 2) (21). Under such conditions, a transient increase in osmolarity of the airway surface liquid is predicted to occur. It is the increase in osmolarity that has been proposed as the *stimulus* causing the airways to narrow in response to exercise (3). This proposal has been supported by (i) *in vitro* studies demonstrating that human lung mast cells release bronchoconstrictor substances when osmolarity increases (22–24) and (ii) *in vivo* studies showing a similar reduction in lung function after inhaling agents with high osmolarity [e.g., an aerosol of a saline solution (25) or a dry powder of mannitol (26, 27)] and after an exercise or a dry air hyperpnea test.

**Figure 2:**
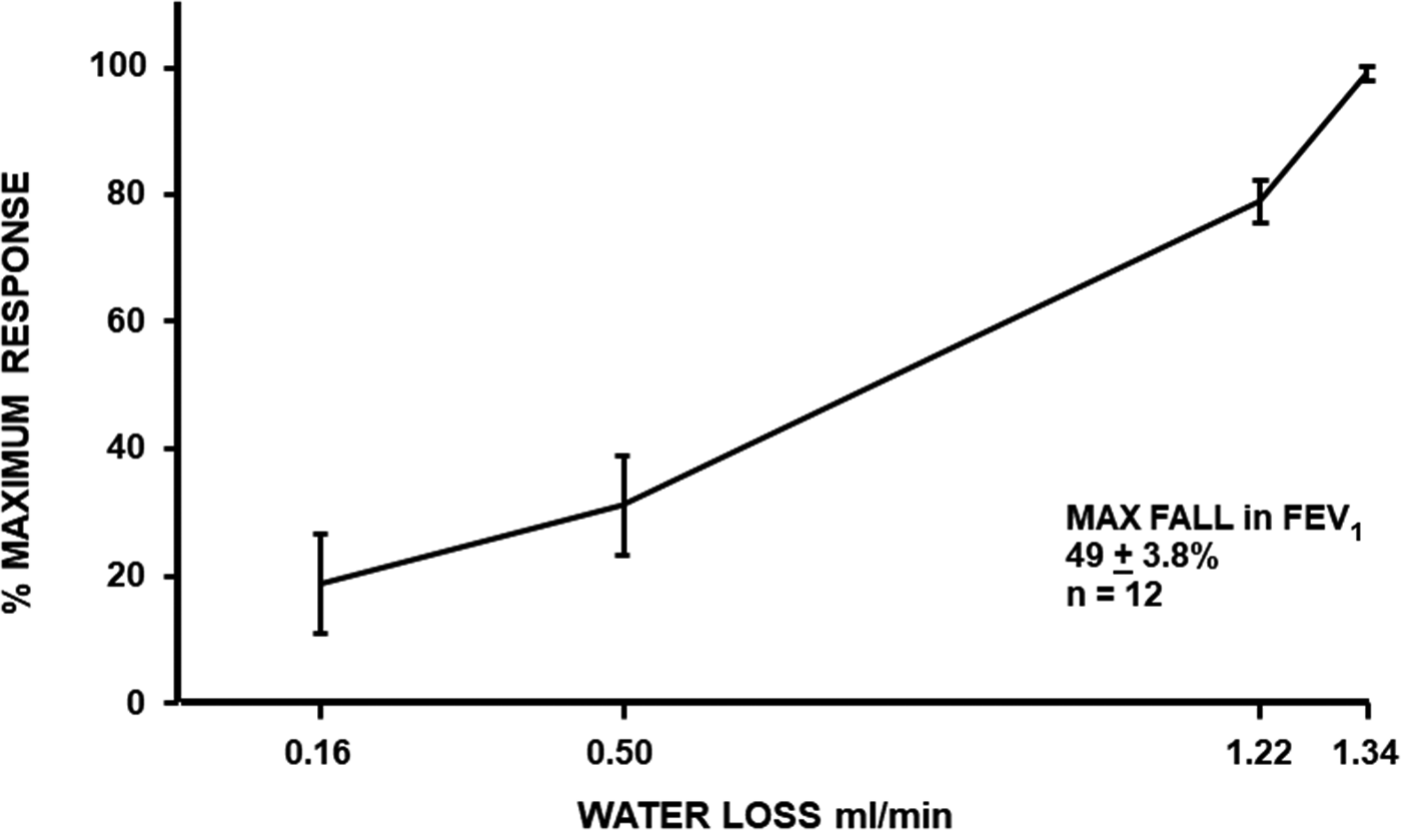
Maximum percentage fall in FEV_1_ in relation to the rate of loss of water from the airways during four exercise tests, each performed while inspiring air of a different condition (hot wet, warm humid, ambient, and medical air) in 12 participants with exercise-induced bronchoconstriction. Based on the data reported by Anderson et al. (21).

The *mechanism* whereby an increase in osmolarity can act as a stimulus for EIB is via the release of inflammatory mediators. The principal source of these mediators, or *biomarkers*, is most likely mast cells situated on or near the airway surface (28) (Figure 3). Further, it has been reported that the density of mast cells per volume is greater in those with EIB relative to normal controls and asthmatics without EIB (29).

**Figure 3:**
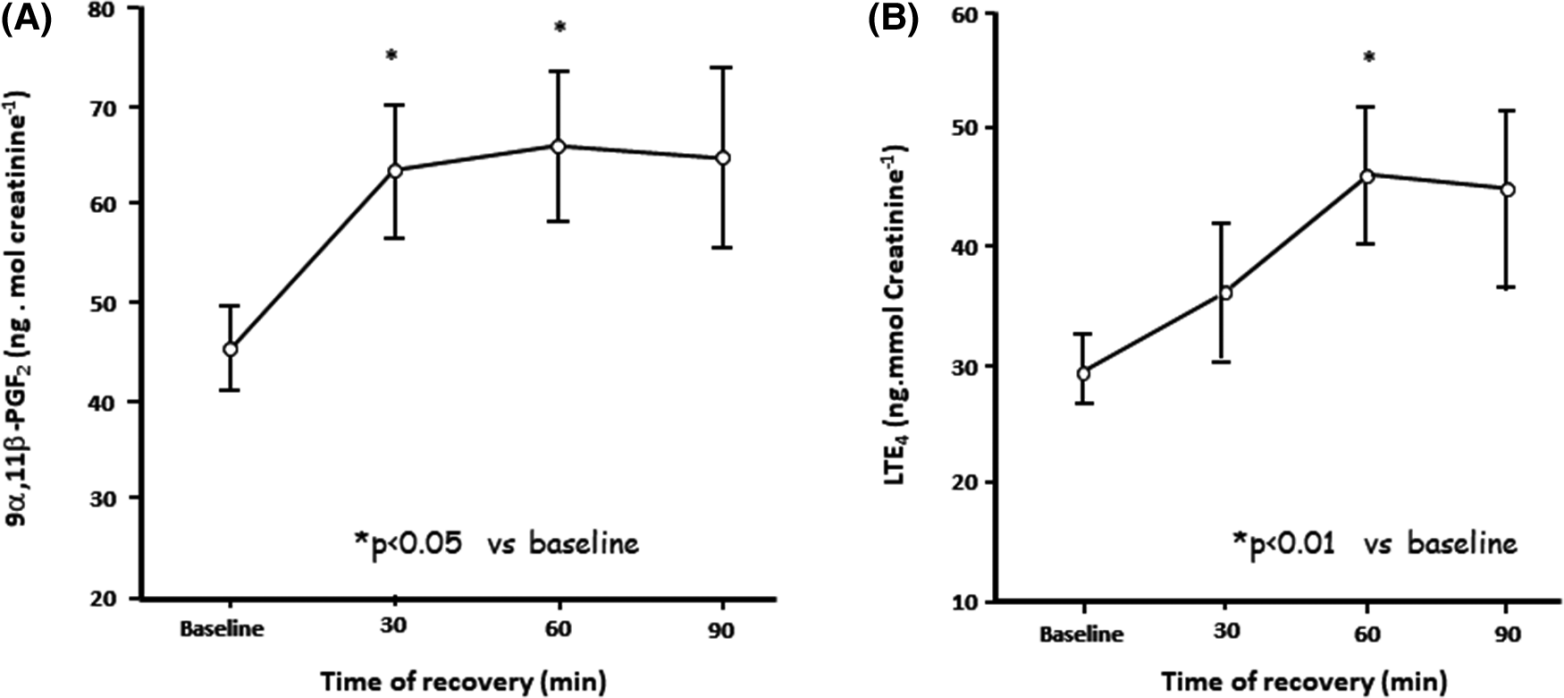
Change in urinary excretion of levels of (**A**) prostaglandin D_2_ metabolite 9 alpha 11 beta prostaglandin F_2_ and (**B**) leukotriene E_4_ levels over 90 min following 6 min of eucapnic voluntary hyperpnea in eight untrained asthmatics. Reproduced from Anderson and Kippelen (18).

Mast cell mediators include histamines, leukotrienes E_4_, and prostaglandins D_2_ (5, 6, 30–32). All these mediators would appear to contribute to the bronchoconstriction induced by hyperpnea of dry air (33). Histamine is however less potent in causing bronchoconstriction than the leukotrienes (34). Originally, mast cell mediators were identified using pharmacological antagonists, which either prevent the release or the action of these mediators (7–9). These mediators are thought to act on bronchial smooth muscle cells to cause contraction and narrowing of the airways, ultimately manifesting as EIB.

To assess the effects of pharmacological agents, both the % fall in FEV_1_ or PEF and the area under the curve over time have been used (7). Histamines are preformed mediators and, as such, are immediately available for release to provoke bronchoconstriction. This is likely why histamine is an important contributor to the maximum % fall in PEF or FEV_1_ after exercise. Because histamines are rapidly metabolized, these are not likely to be important in prolonging the bronchoconstriction due to exercise. Antihistamines do not prevent EIB and are not recommended for treatment; however, the histamine antagonist, loratadine, has been shown to reduce the severity of EIB when taken for 3 days before exercise (8) (Figure 4).

**Figure 4:**
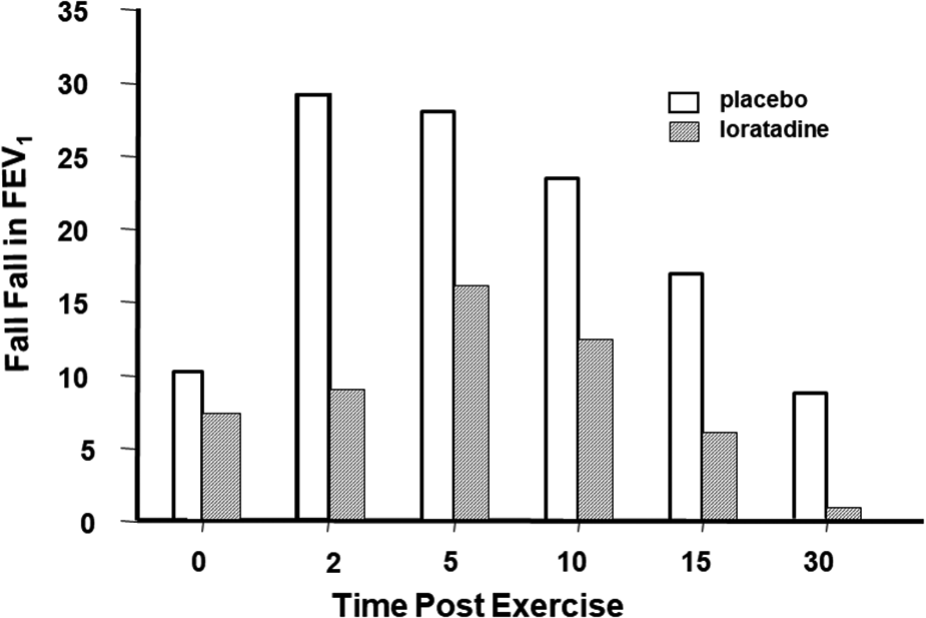
Maximum percentage fall in FEV_1_ at various times following an exercise test in 11 children who had received 10 mg of the antihistamine loratadine or its placebo once daily for 3 days. Drawn from Baki and Orhan (8).

In contrast to histamines, the mast cell mediators, prostaglandin D_2_ and Leukotriene E_4_, are not preformed. For this reason, they are likely to be released from the mast cell later than histamines. Further, unlike histamines, PGD_2_ and LTE_4_ are not rapidly metabolized. Studies using pharmacological antagonists indicate that PGD_2_ and LTE_4_ contribute significantly to the area under the response curve over time. For example the leukotriene antagonist montelukast reduces the severity of EIB, although it does not completely inhibit EIB, as other mediators are also involved. The combination of montelukast and loratadine reduced the concentration of mediators in sputum following exercise, and this was associated with EIB inhibition (28). Sodium cromoglycate (SCG), a mast cell stabilizer, has been reported to inhibit the release of a metabolite of prostaglandin D_2_ in response to hyperpnea with dry air (Figure 5) (10). Another study reported the inhibitory effects of the beta_2_ adrenoceptor agonist terbutaline, given by inhalation in a dose of 500 μg, on mast cell-derived mediator release in EIB (Figure 6) (35). As it had previously been shown that 200 μg of inhaled salbutamol was more effective in preventing EIB than 4 mg of salbutamol taken orally (36), it is likely that a dual action (i.e., relaxation of the airway smooth muscle plus stabilization of mast cells) is responsible for the prophylactic effect of inhaled beta_2_ adrenoceptor agonists on EIB.

**Figure 5:**
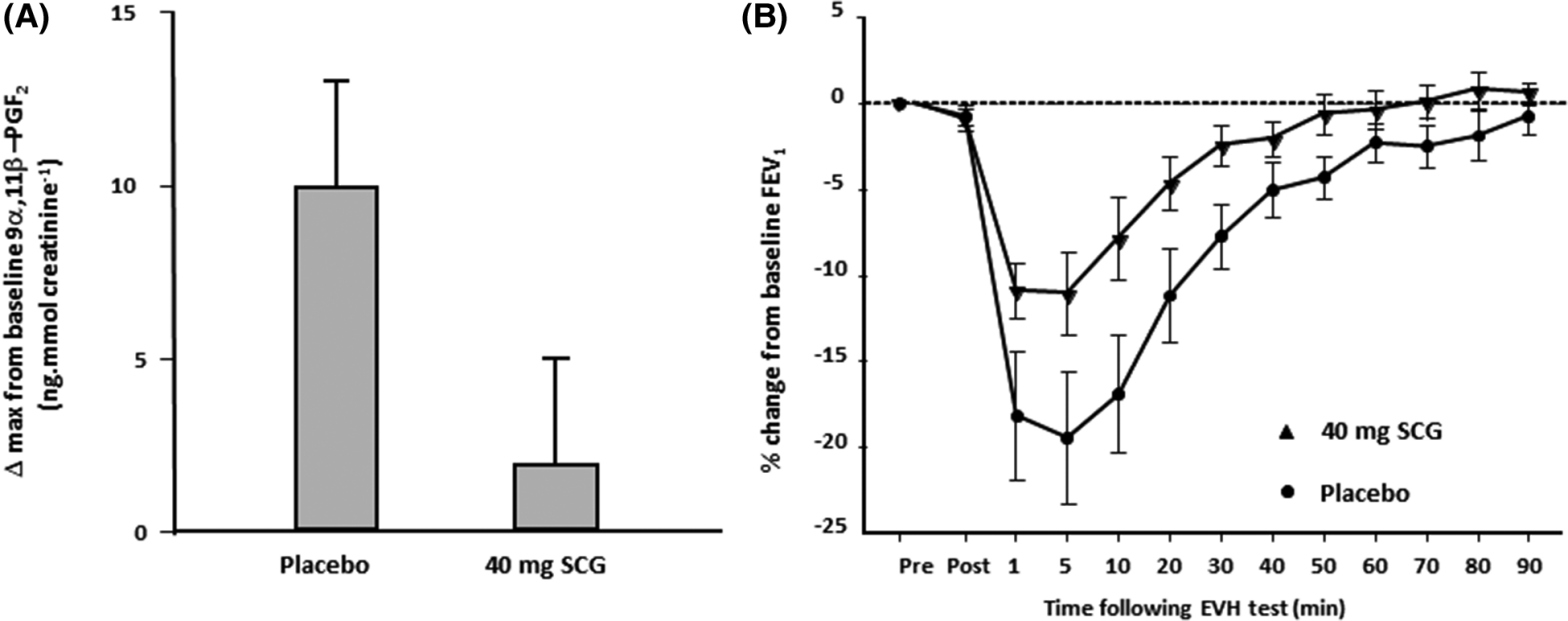
(**A**) Change in the maximum value for 9 alpha, 11 beta prostaglandin F2, a metabolite of prostaglandin D_2_, in 11 athletes following 8 min of eucapnic voluntary hyperpnea with dry air performed 10 min after inhaling 40 mg of SCG and after placebo. Reproduced from Anderson and Kippelen (18). (**B**) Percentage change in FEV_1_ from baseline over 90 min in 11 athletes following 8 min of eucapnic voluntary hyperpnea with dry air performed 10 min after inhaling 40 mg of SCG (triangles) and after placebo (closed circles). Reproduced from Anderson and Kippelen (18).

**Figure 6:**
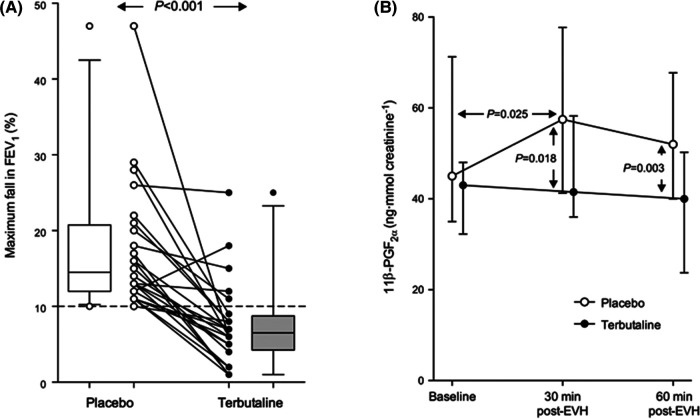
(**A**) Individual values for the maximum fall in FEV_1_ in 27 athletes in response to 8 min of eucapnic voluntary hyperpnea with dry air performed 15 min after inhaling 500 μg of terbutaline sulfate and its placebo. The broken line identifies the threshold for a positive EIB test (i.e., a fall in FEV_1_ of at least 10%). Reproduced from Simpson et al. (35). (**B**) Median values (and interquartile range) for the urinary concentration of 11 *β*-PGF_2_*_α_* at baseline and 30 and 60 min post-eucapnic voluntary hyperpnea with dry air following inhalation of 500 μg of terbutaline sulfate and its placebo in 18 athletes with EIB. Reproduced from Simpson et al. (35).

In conclusion, inflammatory mediators are released in response to exercise and, in those individuals with asthma, are likely to account for the airway narrowing following exercise. As such, preventative methods for EIB should focus on reducing the stimulus (i.e., hyperosmolarity in the lower airways), inhibiting mediators release, or counteracting the mediators’ effects.
